# Gut microbiota profiling reflects the renal dysfunction and psychological distress in patients with diabetic kidney disease

**DOI:** 10.3389/fendo.2024.1410295

**Published:** 2024-07-15

**Authors:** Qi Li, Suyi Xie, Yali Liu, Wei Yue, Limin Wang, Yi Liang, Yan Chen, Huijuan Yuan, Jiawei Yu

**Affiliations:** ^1^ Heart Center of Henan Provincial People’s Hospital, Department of Cardiology of Central China Fuwai Hospital, Henan Key Laboratory for Coronary Heart Disease Prevention and Control, Central China Fuwai Hospital of Zhengzhou University, Zhengzhou, China; ^2^ Department of Medicine Huddinge, Karolinska Institute, Huddinge, Sweden; ^3^ Li Chiu Kong Family Sleep Assessment Unit, Department of Psychiatry, Faculty of Medicine, The Chinese University of Hong Kong, Hong Kong, Hong Kong SAR, China; ^4^ Department of Nephrology, The 988th Hospital of Joint Logistics Support Forces, People’s Liberation Army, Zhengzhou, Henan, China; ^5^ Department of Endocrinology of Henan Provincial People’s Hospital, Henan Provincial Key Laboratory of Intestinal Microecology and Diabetes Control, People’s Hospital of Zhengzhou University, Henan Provincial People’s Hospital of Henan University, Zhengzhou, China

**Keywords:** gut microbiota, diabetic kidney disease, end-stage renal disease, renal dysfunction, psychological distress

## Abstract

**Background:**

The gut microbiota plays a pivotal role in the development of diabetes and kidney disease. However, it is not clear how the intestinal microecological imbalance is involved in the context of diabetic kidney disease (DKD), the leading cause of renal failure.

**Objectives:**

To elucidate the gut microbial signatures associated with DKD progression towards end-stage renal disease (ESRD) and explore whether they could reflect renal dysfunction and psychological distress.

**Methods:**

A cross-sectional study was conducted to explore the gut microbial signatures of 29 DKD non-ESRD patients and 19 DKD ESRD patients compared to 20 healthy controls. Differential analysis was performed to detect distinct gut microbial alterations in diversities and taxon abundance of DKD with and without ESRD. Renal dysfunction was estimated by urea, creatinine, and estimated glomerular filtration rate. Psychological distress was assessed using the Self-Rating Anxiety Scale, Self-Rating Depression Scale, Hamilton Anxiety Rating Scale, and Hamilton Depression Rating Scale.

**Results:**

Alpha diversity indexes were reduced in DKD patients, particularly those with ESRD. Beta diversity analysis revealed that the gut microbial compositions of DKD patients were different with healthy individuals whereas similar compositions were observed in DKD patients. Taxon differential analysis showed that when compared with the controls, DKD patients exhibit distinct microbial profiles including reduced abundances of butyrate-produced, anti-inflammatory bacteria *Faecalibacterium*, *Lachnospira*, *Roseburia Lachnoclostridium*, and increased abundances of pro-inflammatory bacteria *Collinsella*, *Streptococcus* etc. These distinctive genera presented consistent associations with renal dysfunction, as well as psychological distress, especially in DKD patients.

**Conclusions:**

DKD patients, especially those who have progressed to ESRD, exhibit unique characteristics in their gut microbiota that are associated with both renal dysfunction and psychological distress. The gut microbiota may be a significant factor in the deterioration of DKD and its eventual progression to ESRD.

## Introduction

As reported by GBD 2021 Diabetes Collaborators, the global number of people living with diabetes was estimated to be 529 million in 2021 and projected to be around 1.31 billion by 2050, its prevalence rate is still alarmingly escalating across every age group, ranging from children to older adult (1, 2). Apart from leading to the 8^th^ cause of death and disability worldwide, the socioeconomic burden on healthcare systems has also been aggravated by its multiple comorbidities (1, 3), especially diabetic kidney disease (DKD), the leading cause of renal failure requiring renal replacement therapy (dialysis or transplant) (4). Moreover, the progression of DKD seems irreversible due to limited preventive measures, even though the tight glycemic control, and maintenance of a glycosylated hemoglobin (HbA1c) value lower than 7%, yielded little gain (5). Thus, the importance of early detection and prevention of DKD cannot be overstated.

The human gut microbiota, often referred to as the second brain, contains more complex information than the human host itself. Its composition is highly influenced by host genotypes, environmental factors such as diet and exercise, human physiological states, and various disorders. Accumulating evidence has proved that the interplay among gut microbiota, chronic inflammation, immune dysregulation, and metabolism plays a pivotal role in the occurrence and development of diabetes or kidney disease (6–11), but less is known about DKD. Gut microbes could produce short-chain fatty acids (SCFAs, such as butyrate, propionate, and acetate, protecting against diabetes) through the fermentation of plant fibres (12). They, on the other hand, could also generate uremic toxins (such as *p*-Cresol, phenol, and indole, worsening renal function) through the fermentation of amino acids (13). Even though most findings regarding gut dysbiosis, alterations of the normal composition of gut microbiota, appear to be in chaos, there is at least a consensus on the reduced abundance of commensal or symbiotic microbes (e.g., *Bifidobacterium*, and butyrate-producing genera, including *Roseburia* and *Faecalibacterium*) in both type 2 diabetes and kidney disease (6, 9). However, the performance of several genera remains controversial, just like the *Fusobacterium* presents a positive association with type 2 diabetes, but a negative association with chronic kidney disease (CKD) (6, 14). To date, few studies have explored the role of gut dysbiosis in DKD, which involves declining renal function against a backdrop of hyperglycemia and uremic toxins.- The canagliflozin, a sodium-glucose cotransporter (SGLT) inhibitor for diabetic treatment, exhibits a dual inhibition on both SGLT2 (predominantly located on the proximal tubules of the kidney, responsible for urinary glucose excretion) and SGLT1 (highly expressed in the brush-border membrane in the small intestine, responsible for dietary glucose absorption) (15). Interestingly, it remarkedly reduced plasma levels of uremic toxin (*p*-Cresol sulfate and indoxyl sulfate), accompanied by the increased intestinal levels of SCFAs and altered gut microbial composition in the CKD mice model, and this intestinal effect was also independent of blood glucose levels (15). To evaluate the human kidney-specific elimination for metabolites and uremic toxins, Kikuchi K et al. exploited a combined utilization of untargeted metabolomics and transgenic rats engineered to overexpress the SLCO4C1 in the proximal tubule, the only organic anion transporting polypeptide in human kidneys, they discovered phenyl sulfate, a metabolite derived from gut microbiota, caused albuminuria and damage to podocytes in experimental diabetes models, and predicted the progression of renal dysfunction in diabetic patients (16). When inhibiting the tyrosine phenol-lyase, a bacterial enzyme responsible for the synthesis of phenol from dietary tyrosine before it is metabolized into phenyl sulfate in the liver, the albuminuria in diabetic mice could be reduced, indicating that phenyl sulfate also has great potential to act as a therapeutic target for DKD (16). As reported, DKD patients display distinct gut microbial characteristics, for example, their richness and diversity were lower than the healthy individuals (17) but higher than non-diabetic kidney disease (18). In specific, *Roseburia* was not only significantly decreased in DKD patients (19) but also positively correlated with an estimated glomerular filtration rate (eGFR) and negatively correlated with serum creatinine (CREA) (18). Currently, studies are notably deficient in gut microbial signatures associated with the DKD progression to ESRD. Patients with DKD suffer from reduced quality of life and have an increased risk of morbidity and mortality (20). Beyond reduced quality of life, evidence showed that the prevalence of psychological distress (including anxiety and depression), was up to 40-50% in Chinese DKD (21). It was well-demonstrated that anxiety and depression are closely in connection with the microbial community that lives in the gastrointestinal system (22). When intraperitoneally administrating the *p*-Cresol sulfate or indoxyl sulfate to the unilateral nephrectomized mice, mice developed depression-like, anxiety-like behaviors and cognitive impairment, which could be then alleviated by the uremic toxin adsorbent AST-120 (23, 24). Their relationship under the background of DKD is still far from described. Therefore, this study aims to elucidate the gut microbial signatures associated with DKD progression and to explore whether they could reflect the deterioration of renal function and psychological status.

## Materials and methods

### The cross-sectional study population

This cross-sectional study enrolled 68 individuals dwelling in Central China, Henan province, including 20 healthy controls, 29 DKD without end-stage renal disease (ESRD) patients (DKD non-ESRD) and 19 DKD with ESRD patients (DKD ESRD). From April 2021 to February 2023, eligible patients with DKD were recruited in a clinical observational study (988YY2021060LLSP) overseen by the 988th Hospital of Joint Logistics Support Forces, People’s Liberation Army (Zhengzhou, China). Healthy controls came from another observational study which is registered in the Chinese Clinical Trial Registry (ChiCTR2000029237), which was approved by the Medical Ethics Committee of Henan Provincial People’s Hospital (Zhengzhou, China). Written informed consent was obtained from all subjects and all study procedures complied with the principles of the Declaration of Helsinki. Comprehensive information documented in the electronic medical records at recruitment was collected for each subject, including anthropometric measures, medical and medication history, and routine tests for renal and hepatic function. The overall renal function was assessed by the eGFR, which was calculated using the CKD epidemiology collaboration (CKD-EPI) formula (25).

The inclusion criteria for DKD patients were as follows: 1) age between 18 and 70 years; 2) type 2 diabetes diagnosed by the World Health Organization Criteria (26); 3) DKD defined as the CKD caused by diabetes, mainly characterized by urinary albumin/creatinine ratio (UACR) ≥30 mg/g and/or eGFR < 60 ml/min/1.73m^2^, and duration more than 3 months, which was in line with “2021 Chinese Guidelines for Diagnosis and Treatment of Diabetic Kidney Disease” (27); 4) divided into DKD non-ESRD (eGFR ≥15 ml/min/1.73m^2^) and DKD ESRD groups (eGFR <15 ml/min/1.73m^2^); 5) patients in DKD ESRD group underwent maintenance hemodialysis for more than 3 months, in stable condition and without adjustment of dialyzer, dialysate, time and pattern of dialysis in the past month.

Patients were excluded from this study if they: 1) were type 1 diabetes, gestational diabetes, and other special types of diabetes; 2) had severe gastrointestinal diseases (such as persistent vomiting, constipation, diarrhea) or history of gastrointestinal surgery; 3) had moderate or severe hepatic dysfunction; 4) had other primary and secondary kidney diseases (such as well-diagnosed glomerulonephritis, IgA nephropathy, nephrotic syndrome, hypertensive nephropathy, etc.); 5) were suffering from serious organic diseases (such as cancer, cardiovascular and cerebrovascular diseases, hematopoietic system diseases, etc.); 6) were suffering from infectious diseases (such as tuberculosis and acquired immune deficiency syndrome); 7) were suffering from peptic tract ulcers, urinary tract infections, other endocrine and metabolic diseases (such as hyperthyroidism, polycystic ovary syndrome, etc.) and have received drug treatment in the past 3 months; 8) received antibiotics and probiotics or any other drugs that may affect the intestinal microbiota within the 3 months before inclusion; 9) were pregnant or lactating.

### Psychological distress assessments

In the assessment of psychological distress, a variety of standardized scales and questionnaires are utilized, each designed to measure specific aspects of mental well-being and pathology. The Self-Rating Depression Scale (SDS) and the Self-Rating Anxiety Scale (SAS), composed of 4 dimensions (cognitive, autonomic, motor, and central nervous system symptoms), are short (20-item, with each item rated on a 4-point scale), self-administered questionnaires to evaluate the depression and anxiety levels of a patient (28, 29). For more targeted psychological evaluations, the Hamilton Anxiety Rating Scale (HAM-A) and the Hamilton Depression Rating Scale (HAM-D) are frequently employed (30, 31). HAM-A is designed to assess the severity of anxiety symptoms, considering both psychological and somatic symptoms associated with anxiety. HAM-D, on the other hand, is intended to quantify the severity of depression and is one of the oldest and most well-known rating scales for depression. It evaluates mood, feelings of guilt, suicide ideation, insomnia, agitation or retardation, anxiety, weight loss, and somatic symptoms. All of them were assessed by trained researchers.

### Cross-sectional stool sample collection

Fresh feces in the morning were collected using sterile spoons and placed into 8 mL sterilized freezer tubes. All subjects received education to avoid urine contamination during collection. Then the stool samples were stored at -80°C immediately.

### PCR amplification and 16S rRNA sequencing

Following the manufacturer’s instructions of the E.Z.N.A. ^®^ Stool DNA Kit (Omega Bio-Tek, Inc., GA), genomic DNA was extracted from feces swabs. After isolation, DNA integrity and concentration were assessed by agarose electrophoresis and NanoDrop (Thermo Scientific, USA) microvolume spectrophotometer, respectively. PCR was performed with the following primers: forward, 5’-CCTACGGGNGGCWGCAG-3’; and reverse, 5’-GACTACHVGGGTATCTAATCC-3’, which correspond to positions 341 to 805 in the Escherichia coli 16S rRNA gene, to amplify the V3–V4 region of each sample. Subsequent amplicon sequencing was performed by Shanghai Mobio Biomedical Technology Co., Ltd. on a MiSeq platform (Illumina, San Diego, CA, USA).

### Sequencing data analysis

Sequences were analyzed using QIIME2 version 2023.5. After importing the raw FASTQ data, the non-biological parts of the sequences (such as primers and adapters) were removed using the “cutadapt” plugin of QIIME2. Sequences were then truncated with DADA2 and further filtered and denoised, after which chimaeras were removed. Next, the sequences were merged to obtain the abundance and representative sequences of amplicon sequence variants (ASVs). Representative sequences for ASVs were built into a phylogenetic tree using the core-metrics-phylogenetic pipeline in QIIME2, after which taxonomy was assigned using the SILVA database (release 138). All samples for gut microbiota analysis were randomly subsampled to equal depths of 10956 reads, before stool microbiome analysis using QIIME2 diversity plugins.

### Statistical analysis

Average values were expressed as median with Quantile 1 (Q1) and Quantile 3 (Q3) for continuous variables and as numbers with percentages (%) for categorical variables, where appropriate. Differences between groups were compared using the Kruskal-Wallis H test with Dunn *post hoc* test and Chi-square test.

Taxa not present in at least one sample were pruned before analysis. To describe the distribution of abundances in each sample, alpha diversity was analyzed using Chao1, Shannon, Pielou and Simpson indexes to evaluate richness, diversity, evenness, and dominance, respectively. To quantify (dis-)similarities between samples, beta diversity was measured by Bray-Curtis and Jaccard indexes, Unweighted and Weighted UniFrac distances, which were reflected by the Principal Coordinate Analysis (PCoA) method. Permutational Multivariate Analysis of Variance test (PERMANOVA, 999 tests) was performed to estimate the differences in observed community composition between groups.

To find the distinctive genera (detailed descriptions are shown in **Supplementary Table 1**), raw counts were transformed using the centered log-ratio (clr) method on the genus level of core gut microbiota (detection rate: 0.1%, prevalence threshold: 5%) and differences between groups were compared using Kruskal-Wallis H test with Dunn *post hoc* test. The MaAsLin2 method was used to perform pairwise comparisons among three groups (32). To examine if these taxa remained significantly different, two models were utilized: one including the grouping factor alone, the other one adjusting for age, sex and BMI. The Spearman correlation method with or without the adjustment of age, sex and BMI was chosen to analyze the associations between the clr-transformed counts of each genus and the indicators of renal function (UREA, CREA, eGFR) or psychological distress (SAS, SDS, HAM-A, HAM-D) among the overall participants, healthy control, or patients with DKD. *P* values were corrected using the Bonferroni method. R software (v4.3.2) was adopted for data statistics.

## Results

### Clinical characteristics of DKD patients with or without ESRD

Clinical characteristics of DKD patients with or without ESRD, compared to healthy controls

68 individuals participated in this study, including 20 healthy controls, 29 DKD non-ESRD, and 19 DKD ESRD. The clinical characteristics and laboratory findings of these groups are summarized in **Table 1**.

**Table 1:** Clinical characteristics of patients with diabetic kidney disease and healthy controls.

Characteristics	Healthy control (n = 20)	DKD non-ESRD (n = 29)	DKD ESRD (n = 19)	*P*
Age, years	51.5 (43.5-59.8)	60.0 (55.0-65.0)	48.0 (38.5-66.0)	0.09
Sex, percent female	10 (50.0)	9 (31.0)	6 (31.6)	0.34
BMI, kg/m^2^	25.8 (22.4-28.9)	27.1 (23.2-29.4)	22.4 (20.9-24.8) ^$^	1.14 x 10^-2^
WBC, 10^9^/L	5.4 (4.9-6.1)	6.5 (5.2-8.0)	5.0 (3.7-5.4) ^$^	8.76 x 10^-4^
CRP, mg/L	1.7 (1.0-3.0)	4.1 (2.5-5.3) ^#^	4.8 (4.0-6.3) ^&^	1.60 x 10^-4^
RBC, 10^12^/L	4.2 (4.0-4.6)	4.2 (4.0-4.6)	3.1 (2.6-3.5) ^$&^	3.91 x 10^-7^
PLT, 10^12^/L	189.0 (169.2-232.2)	203.0 (172.0-262.0)	170.0 (150.5-206.5)	0.12
ALT, g/L	26.5 (18.0-47.0)	24.0 (13.0-47.0)	17.0 (10.0-27.5)	0.11
AST, g/L	26.0 (18.0-48.5)	22.0 (16.0-47.0)	15.0 (11.5-25.0) ^&^	1.61 x 10^-2^
UMAC, mg/L	/	162.8 (86.4-475.1)	/	/
UACR, mg/g	/	311.5 (86.4-841.6)	/	/
UA, µmol/L	374.0 (312.8-412.5)	278.0 (251.0-416.0)	435.0 (263.0-532.5)	0.09
UREA, mmol/L	4.9 (4.1-5.2)	4.6 (3.5-5.2)	18.1 (14.3-24.1) ^$&^	1.06 x 10^-9^
CREA, µmol/L	58.5 (47.8-66.2)	62.0 (55.0-75.0)	784.0 (642.5-960.0) ^$&^	7.48 x 10^-10^
eGFR, ml/min/1.73m^2^	104.0 (95.4-108.0)	92.4 (75.2-99.5)	4.8 (3.8-6.1) ^$&^	3.61 x 10^-10^
SAS	44.0 (37.5-52.5)	43.0 (38.0-52.0)	55.0 (41.2-57.5)	0.05
SDS	42.0 (36.0-50.5)	41.0 (35.0-47.0)	54.5 (50.0-59.5) ^$&^	2.01 x 10^-4^
HAM-A	5.0 (4.0-6.0)	5.0 (4.0-7.0)	7.5 (6.0-9.0) ^$&^	4.32 x 10^-3^
HAM-D	14.0 (11.5-16.0)	13.0 (12.0-15.0)	18.5 (16.0-20.0) ^$&^	5.10 x 10^-5^

ALT, alanine transaminase; AST, aspartate aminotransferase; BMI, body mass index; CRP, C-reactive protein; CREA, creatinine; DKD, Diabetic Kidney Disease; eGFR, estimated glomerular filtration rate; ESRD, End-Stage Renal Disease; HAM-A, Hamilton Anxiety Rating Scale; HAM-D, Hamilton Depression Rating Scale; PLT, platelet; RBC, red blood cells; SAS, Self-Rating Anxiety Scale; SDS, Self-Rating Depression Scale; UACR, urinary albumin/creatinine ratio; UMAC, urinary microalbumin concentration; WBC, white blood cells. Continuous variables were expressed as median (quantile 1-quantile 3) and categorical variables as numbers (percentage). The Kruskal-Wallis H test with the Dunn post hoc test and Chi-square test were used to compare the differences between groups. ^#^P < 0.05, DKD non-ESRD vs. healthy control; ^$^P < 0.05, DKD ESRD vs. DKD non-ESRD; ^&^P < 0.05, DKD ESRD vs. healthy control.

The “/” indicates not applicable.

In the biochemical analysis, aspartate aminotransferase (AST) levels were significantly lower in the DKD ESRD group compared to healthy controls, with median values of 15.0 U/L (Q1-Q3: 11.5-25.0) versus 26.0 U/L (Q1-Q3: 18.0-48.5), respectively (*P* < 0.05). Furthermore, the body mass index (BMI) of DKD ESRD patients was reduced at a median value of 22.4 kg/m² (Q1-Q3: 20.9-24.8) compared to healthy controls with a median BMI of 27.1 kg/m² (Q1-Q3: 23.2-29.4) (*P* < 0.01). White blood cell (WBC) counts were also lower in DKD ESRD patients (median: 5.0 x 10^9^/L, Q1-Q3: 3.7-5.4) compared to DKD non-ESRD patients (median: 6.5 x 10^9^/L, Q1-Q3: 5.2-8.0) (*P <* 0.001). In contrast, C-reactive protein (CRP) levels were higher in both DKD groups when compared to controls, with median values of 4.8 mg/L (Q1-Q3: 4.0-6.3) for ESRD patients and 4.1 mg/L (Q1-Q3: 2.5-5.3) for non-ESRD patients against 1.7 mg/L (Q1-Q3: 1.0-3.0) for healthy individuals (*P* < 0.0001 for ESRD and *P* < 0.05 for non-ESRD, respectively). DKD ESRD patients exhibited significantly lower red blood cell (RBC) counts (median 3.1 x 10¹²/L, Q1-Q3: 2.6-3.5) compared to healthy controls (median: 4.2 x 10¹²/L, Q1-Q3: 4.0-4.6) and DKD non-ESRD patients (median: 4.2 x 10¹²/L, Q1-Q3: 4.0-4.6), with both *P*-values less than 0.0001.

Regarding renal function, UREA and CREA levels were markedly higher and eGFR was dramatically lower in DKD ESRD patients (median UREA: 18.1 mmol/L, Q1-Q3: 14.3-24.1; median CREA: 784.0 µmol/L, Q1-Q3: 642.5-960.0; median eGFR: 4.8 ml/min/1.73m^2^, Q1-Q3: 3.8-6.1), compared to both healthy controls and DKD non-ESRD patients, with all *P*-values less than 0.0001.

Psychological distress, as measured by the SAS, SDS, HAM-A, and HAM-D, was higher in the DKD ESRD group compared to the control and non-ESRD DKD groups. Although the SAS score did not display statistically significant differences in the pairwise comparisons, there was an obvious increase in the SDS score (*P* < 0.01). HAM-A and HAM-D scores were higher in DKD ESRD patients (median HAM-A: 7.5, Q1-Q3: 6.0-9.0, *P* < 0.01; median HAM-D: 18.5, Q1-Q3: 16.0-20.0, *P* < 0.001) when compared to both healthy controls and DKD non-ESRD patients.

In summary, DKD ESRD patients demonstrated distinct biochemical and psychological profiles, characterized by lower AST, BMI, and WBC counts, worse renal function, and higher psychological scales, compared to healthy controls and DKD non-ESRD patients.

### Gut microbiota architecture of DKD about renal dysfunction and psychological distress

**Figure 1:**
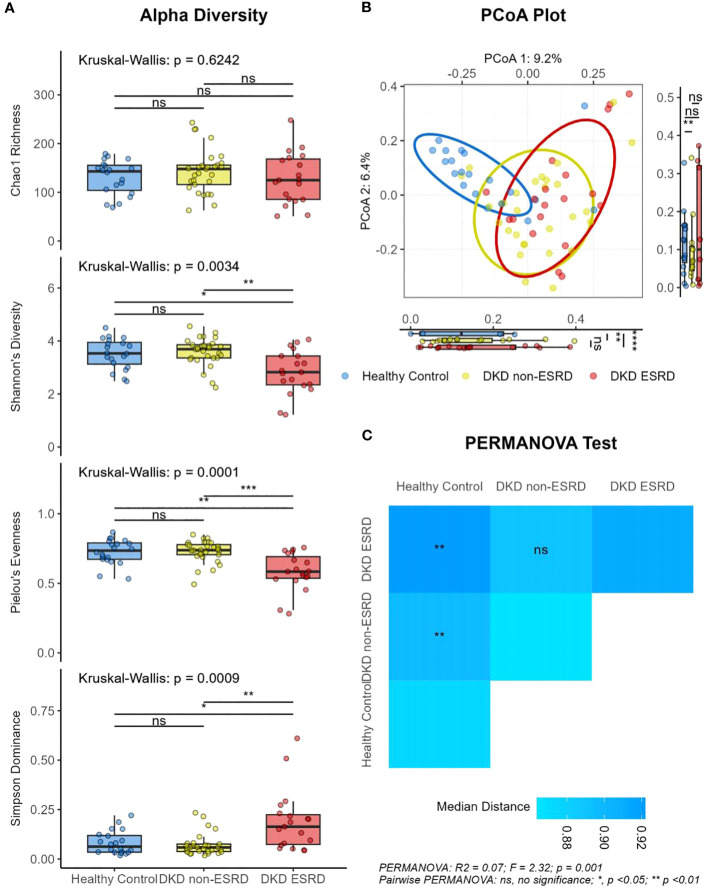
Differential diversity of gut microbiota in patients with diabetic kidney disease and healthy controls. **(A)** Alpha diversity. **(B)** PCoA plot. **(C)** PERMANOVA test results. DKD, Diabetic Kidney Disease; ESRD, End-Stage Renal Disease. Beta diversity analyses were based on the Bray-Curtis distance of gut microbiota. Kruskal-Wallis H test with Dunn post hoc test was used to compare the differences between groups. ns, no significance; *, *P <* 0.05; **, *P <*0.01; ***, *P<*0.001; ****, *P <* 0.001.

The alpha diversity analysis, presented in **Figure 1A**, showed no significant differences in Chao1 richness among the three groups (median: 143.0 vs. 148.0 vs. 125.0; *P* > 0.05). However, when set against both the control group and the DKD non-ESRD patients, DKD ESRD patients demonstrated lower Shannon diversity (median: 3.5 vs. 3.7 vs. 2.8; *P* < 0.01), reduced Pielou’s evenness (median: 0.7 vs. 0.7 vs. 0.6; *P* < 0.001), and greater Simpson dominance (median: 0.1 vs. 0.1 vs. 0.2; *P* < 0.001), suggesting a less diverse and more uneven microbial community.

On the other hand, beta diversity analysis, assessed by PCoA, revealed a clear separation in the composition of the gut microbiota of DKD patients from that of the control subjects, indicating distinct microbial community structures. This pattern was consistently observed in the ordination plots based on various distance metrics, including Bray-Curtis (**Figure 1B**), Jaccard (**Supplementary Figure 1**), Unweighted UniFrac (**Supplementary Figure 2**), and Weighted UniFrac distances (**Supplementary Figure 3**). PERMANOVA tests based on the Bray-Curtis dissimilarity metric indicated that the gut microbial dissimilarity among all individuals was not influenced by the confounders (age, sex, BMI). Instead, the differences could be substantially associated with the classification of the subjects into the groups based on health status (healthy, DKD with or without ESRD, R^2^ = 0.07, *P* < 0.01, **Figure 1C**), UREA (R^2^ = 0.02, *P* < 0.05), CREA (R^2^ = 0.03, *P* < 0.01) or eGFR (R^2^ = 0.03, *P* < 0.01), as well as the SDS (R^2^ = 0.02, *P* < 0.05), HAM-A (R^2^ = 0.02, *P* < 0.01), or HAM-D (R^2^ = 0.02, *P* < 0.05) scores.

### Gut microbiota alterations in diabetic kidney disease at the genus level

**Figure 2:**
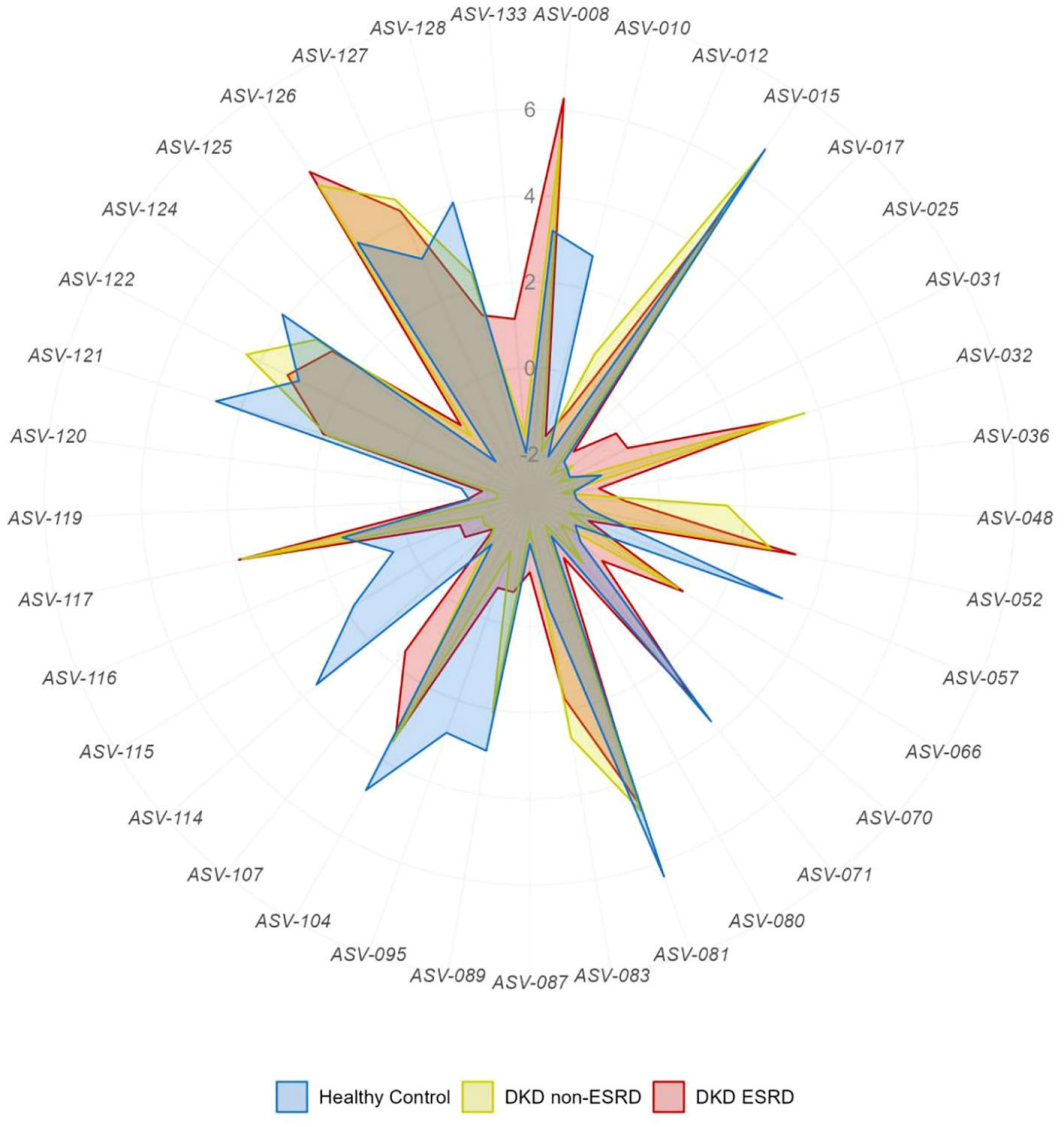
Differential analysis of gut microbiota general abundances in patients with diabetic kidney disease and healthy controls. DKD, Diabetic Kidney Disease; ESRD, End-Stage Renal Disease. Raw counts were transformed using centered log-ratio (clr) method on the genus level of gut microbiota. The detailed description of these genera was shown in **Supplementary Table 1** and the corresponding statistical results using Kruskal-Wallis H test with Dunn post hoc test were shown in **Supplementary Table 2**.

**Figure 3:**
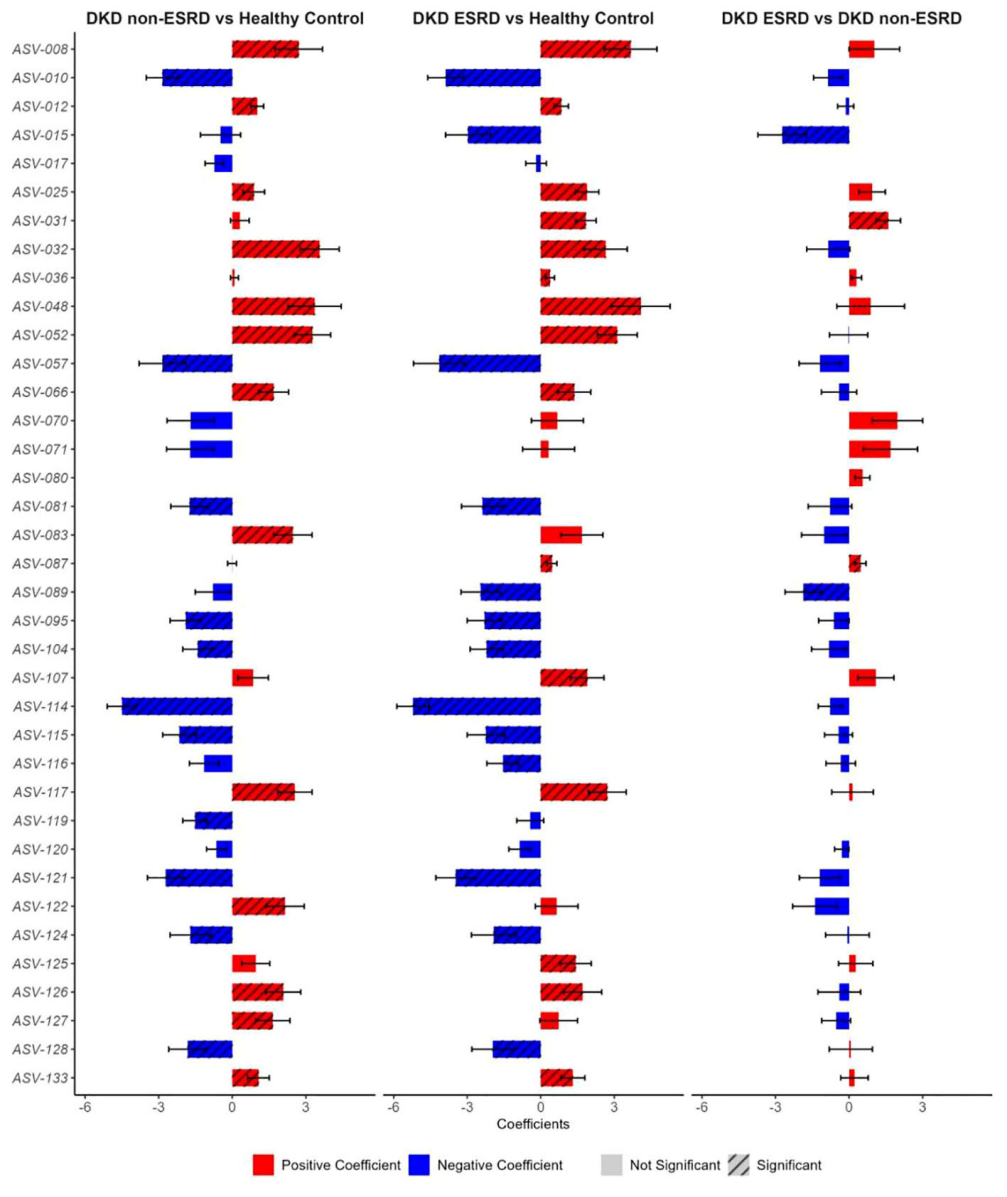
Pairwise comparisons of distinctive gut microbial genera among patients with diabetic kidney disease and healthy controls. BMI, body mass index, DKD, Diabetic Kidney Disease, ESRD, End-Stage Renal Disease. MaAsLin2 method with the adjustment of age, sex, and BMI was applied to calculate the pairwise comparisons of distinctive gut microbial genera among three groups. The positive coefficients were shown in red, whereas those negative coefficients were shown in blue. The bars with or without black slant lines indicated the significant or non-significant different genera. The detailed description of these genera was shown in **Supplementary Table 1** and the corresponding statistical results using MaAsLin2 were shown in **Supplementary Table 3**.

In the differential analysis of the gut microbiome data, we observed a distinct feature of the gut microbiota in patients with DKD at the genus level (**Figure 2**). Detailed information can be found in **Supplementary Table 2**. In DKD patients, there was notable decrease in the relative abundances of specific ASVs including *ASV-010 (Parasutterella), ASV-057 (Dialister), ASV-081 (Faecalibacterium), ASV-095 (UCG-003), ASV-104 (Lachnospiraceae uncultured), ASV-114 (Lachnospira), ASV-121 (Roseburia), ASV-128 (Lachnoclostridium)*, and increased abundances of *ASV-012 (Neisseria), ASV-032 (Collinsella), ASV-052 (Streptococcus), ASV-117 (Eubacterium-hallii group), ASV-126 (Blautia)*. In comparison to the other two groups, DKD non-ESRD patients showed depletion in *ASV-119 (Eubacterium-xylanophilum-group)*, whereas DKD ESRD patients were depleted in ASV-015 (*Bacteroides*) but enriched in *ASV-025 (Coriobacteriales-Incertae-Sedis uncultured), ASV-031 (Adlercreutzia)*. **Figure 3**; **Supplementary Table 3** illustrate that the differences at the genus level within the DKD cohorts remain consistent, even when accounting for potential confounders like age, sex and BMI. These findings suggest that the observed alterations in the gut microbiota composition are independently associated with the DKD condition.

### The distinctive gut microbial genera reflect renal dysfunction and psychological distress

**Figure 4:**
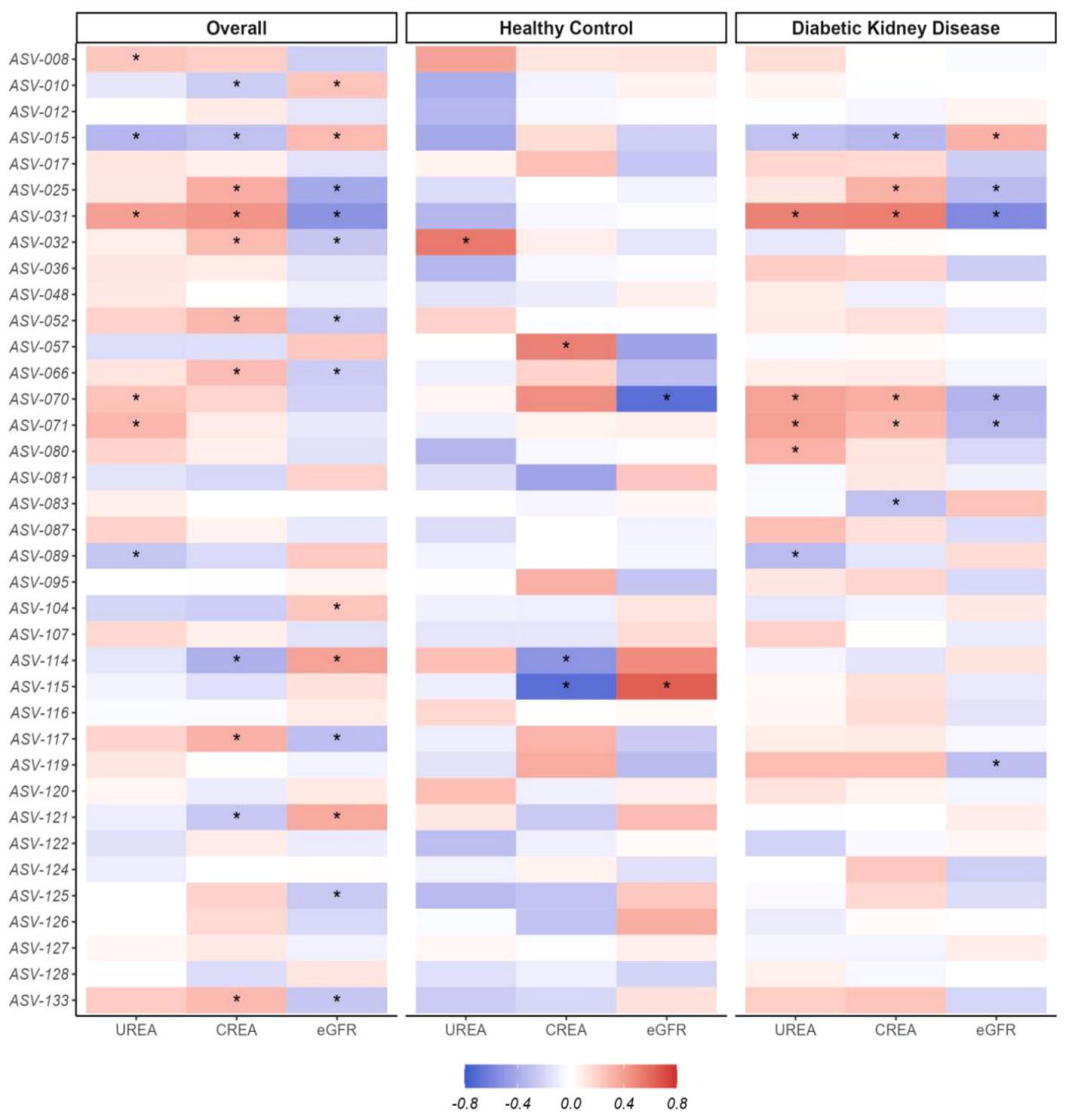
Correlation patterns of the distinctive gut microbial genera and the renal dysfunction. BMI, body mass index; CREA, creatinine, DKD, Diabetic Kidney Disease, eGFR, estimated glomerular filtration rate. eGFR was calculated using CKD-EPI formula The Spearman correlation method with the adjustment of age, sex, and BMI was applied to calculate the associations between the centered log-ratio (clr) transformed counts of each genus and the renal function indicators (UREA, CREA, eGFR) among the overall participants, healthy control, or patients with diabetic kidney disease, *P <0.05. The detailed description of these genera was shown in **Supplementary Table 1** and the corresponding statistical results using Spearman were shown in **Supplementary Table 4**.

**Figure 5:**
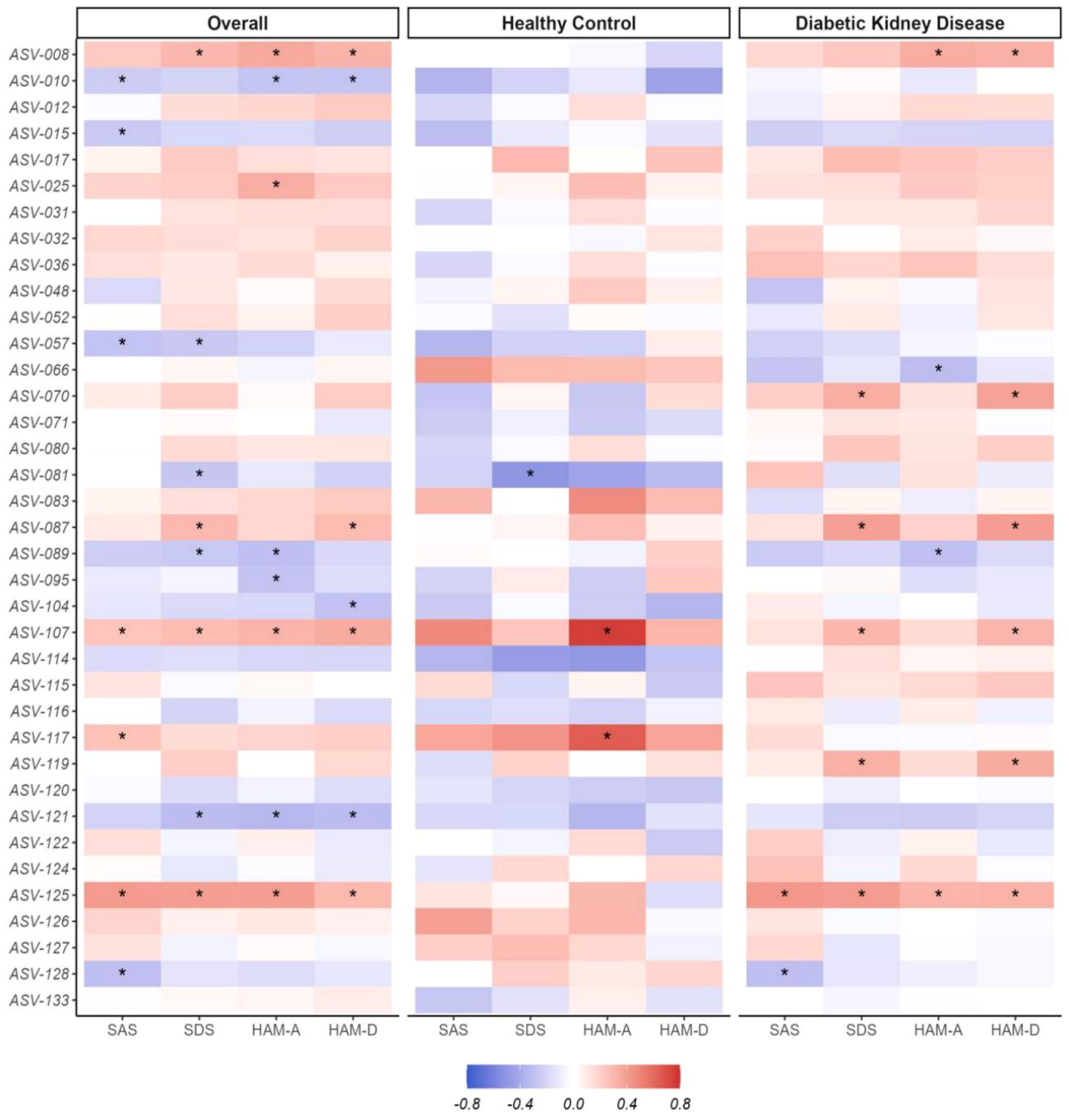
Associations between the distinctive gut microbial genera and the psychological distress. BMI, body mass index; DKD, Diabetic Kidney Disease, HAM-A, Hamilton Anxiety Rating Scale, HAM-D, Hamilton Depression Rating Scale, SAS, Self-Rating Anxiety Scale, SDS, Self-Rating Depression Scale. The Spearman correlation method with the adjustment of age, sex, and BMI was applied to calculate the associations between the centered log-ratio (clr) transformed counts of each genus and the psychological distress (SAS, SDS, HAM-A, HAM-D) among the overall participants, healthy control, or patients with diabetic kidney disease. *P <0.05. The detailed description of these genera was shown in **Supplementary Table 1** and the corresponding statistical results using Spearman were shown in **Supplementary Table 4**.

To elucidate the relationship between gut microbial composition and renal dysfunction or psychological distress, initial analyses were conducted on the entire cohort. These associations accounted for age, sex, and BMI as potential confounding variables. We observed that the associations of DKD-associated alterations in gut microbiota with eGFR were inversely related to their associations with serum UREA and CREA levels, as shown in **Figure 4**. Additionally, there were consistent relationships between these gut microbial genera and psychological scales, including SAS, SDS, HAM-A, and HAM-D, detailed in **Figure 5**. Considering the cross-sectional design of this study may not accurately reflect the broader population and recognize subtle differences in the absolute abundance of specific microbial genera between DKD patient groups, we combined the DKD patients with or without ESRD together and further analyzed these associations in healthy controls and DKD patients separately. As expected, the associations of microbial composition with renal or mental status in healthy controls were not exactly what they presented in DKD patients (**Figures 4**, **5**). For example, the negative associations of *ASV-015 (Bacteroides*), *ASV-031* (*Adlercreutzia*), *ASV-070* (*CAG-352*), and *ASV-071* (*Ruminococcus*) with renal function, as well as the positive associations of *ASV-125* (*Ruminococcus-gauvreauii-group*) with the anxiety and depression severities existed in DKD patients. Detailed information can be found in **Supplementary Table 4**.

## Discussion

This study investigated the role of gut microbiota in the progression of DKD and its transition to ESRD. The results indicate that DKD patients, particularly those with ESRD, have different gut microbiota characteristics compared to healthy controls. These findings add to the growing body of evidence suggesting that gut microbiota may play a crucial role in the development and progression of DKD, a significant health concern given the rising prevalence of diabetes worldwide.

Our clinical characteristics analysis demonstrated that DKD patients with ESRD had lower levels of AST, BMI, and WBC, but higher levels of CRP, UREA, CREA, SDS, HAM-A, and HAM-D, compared to controls and DKD non-ESRD patients. Our findings are consistent with previous findings that anxiety and depression are common comorbidities of diabetes, which are associated with reduced quality of life and a higher risk of DKD and premature death (21). These results suggest an association between DKD progression and changes in these parameters, indicating the potential role of inflammation, metabolic abnormalities, and psychological stress on the progression of DKD, which aligns with previous findings (33).

Interestingly, our analysis of gut microbiota diversity revealed a decrease in Shannon diversity and Pielou’s evenness, along with an increase in Simpson dominance in DKD ESRD patients. This suggests an imbalance in the gut microbiota or dysbiosis, which may have implications for disease progression. Additionally, beta diversity analysis indicated a distinct clustering of gut microbiota in DKD patients, separating them from the controls. Such differences in gut microbiota composition may impact the host’s metabolic profile, potentially contributing to the pathogenesis of DKD. At the genus level, we noted changes in microbial composition tied to DKD. There was a decreased abundance of genera such as *Parasutterella*, *Dialister*, and *Faecalibacterium*. *Dialister*, in particular, has been highly linked with concentrations of uremic toxins like indoxyl sulfate and *p*-Cresyl sulfate, both of which have known nephrotoxic effects (34). *Faecalibacterium*, especially the *species F. prausnitzii*, is recognized for its anti-inflammatory properties and production of SCFAs (35), which may offer protective effects against CKD progression. Furthermore, we observed a decline in members of the *Lachnospiraceae* family, including *Lachnospira* and *Roseburia*. These taxa are significant contributors to the overall production of SCFAs in the gut. A reduction in their abundance could decrease SCFA levels, potentially leading to increased intestinal permeability and inflammation, known risk factors for exacerbating CKD (36, 37). Intriguingly, *Adlercreutzia* was consistently increased in DKD patients, regardless of ESRD status. Our finding was aligned with previous findings: *Adlercreutzia* was enriched in CKD patients and positively associated with TMAO (38). In addition, *Adlercreutzia* was positively correlated with plasma *p*-Cresyl level and its increased abundance in renal failure mice could be reduced by the microbial enzymatic reduction of *p*-Cresyl (16). Interestingly, in the CKD mice model, SGLT2 inhibitor reduced the plasma levels of uremic toxin and increased SCFA production, while the abundance of *Adlercreutzia* was not altered (15). This suggests an unresolved pathway involving phenol-producing bacteria and DKD. Previous studies suggested the other microbial metabolite, 4-ethylphenol, metabolized from tyrosine can alter brain activity and increase anxiety-like behavior in mice (39, 40). This genus has been implicated in the relief of depressive symptoms, with evidence suggesting that antidepressant treatments may elevate *Adlercreutzia* abundance (41). The precise role of *Adlecreutzia* in DKD and its involvement in uremic toxin production remains uncertain, but its consistent elevation across studies calls for more in-depth investigation.

Several distinctive genera identified in this study have been associated with lipid metabolism and atherosclerosis (ACS). For instance, enzymes involved in bile acid oxidation have been found in *Blautia (* 42) and *Lachocolstridium* promoting ACS (43). Targeting gut microbiota might provide novel strategies to ease the high burden of dyslipidemia and cardiovascular mortality in CKD, as well as delay the transition to ESRD (44). DKD patients are malnourished since they have reduced absorption of nutrients and waste, which would alter the gut microbiota compositions. However, the exact mechanisms through which these bacteria may contribute to DKD progression require further investigation.

This study has several limitations that warrant discussion. Firstly, the modest sample size provides preliminary insights into the gut microbial characteristics of DKD, yet larger cohorts are necessary to corroborate these initial findings. Secondly, the cross-sectional nature of our study means we can observe differences in the microbial compositions of patients with DKD and varying renal functions, but we cannot ascertain causality or predict disease progression. To address this, a longitudinal study design, incorporating multiple time points for assessing gut microbiota, would be invaluable in tracing the trajectory of the disease. Lastly, while diet plays a crucial role in shaping the gut microbiota of both patients and healthy individuals, it was not evaluated in our investigation. Given that patients with kidney diseases often experience reduced appetite and dietary modifications, which in turn can alter gut microbiota composition, future research should integrate dietary assessments. We recommend that our colleagues consider the significant impact of diet on both the pathophysiology and psychopathology of DKD, from study conception through to data analysis.

In conclusion, our study provides valuable insights into the distinctive gut microbial characteristics associated with DKD progression. These findings underscore the potential of gut microbiota as a target for early detection and preventive measures for DKD. However, additional longitudinal and mechanistic studies are warranted to further understand the role of gut microbiota in DKD and to validate these results in different populations and clinical settings.

## Data availability statement

The original contributions presented in the study are included in the article/**Supplementary Material**, further inquiries can be directed to the corresponding author/s.

## Ethics statement

The studies involving humans were approved by the 988th Hospital of Joint Logistics Support Forces, People’s Liberation Army (988YY2021060LLSP) and the Medical Ethics Committee of Henan Provincial People’s Hospital (ChiCTR2000029237). The studies were conducted in accordance with the local legislation and institutional requirements. The participants provided their written informed consent to participate in this study.

## Author contributions

QL: Conceptualization, Data curation, Formal analysis, Methodology, Validation, Visualization, Writing – original draft, Writing – review & editing. SX: Conceptualization, Data curation, Formal analysis, Methodology, Validation, Visualization, Writing – original draft, Writing – review & editing. YaL: Supervision, Validation, Writing – review & editing. WY: Supervision, Validation, Writing – review & editing. LW: Supervision, Validation, Writing – review & editing. YiL: Supervision, Validation, Writing – review & editing. YC: Supervision, Validation, Writing – review & editing. HY: Data curation, Investigation, Project administration, Resources, Supervision, Writing – review & editing. JY: Conceptualization, Funding acquisition, Investigation, Project administration, Resources, Supervision, Validation, Writing – review & editing.
