# Supplementary figures and images for: Gut microbiota profiling reflects the renal dysfunction and psychological distress in patients with diabetic kidney disease
Source: Front Endocrinol (Lausanne). 2024 Jul 15;15:1410295. doi: 10.3389/fendo.2024.1410295 (PMC11284015; doi:10.3389/fendo.2024.1410295)

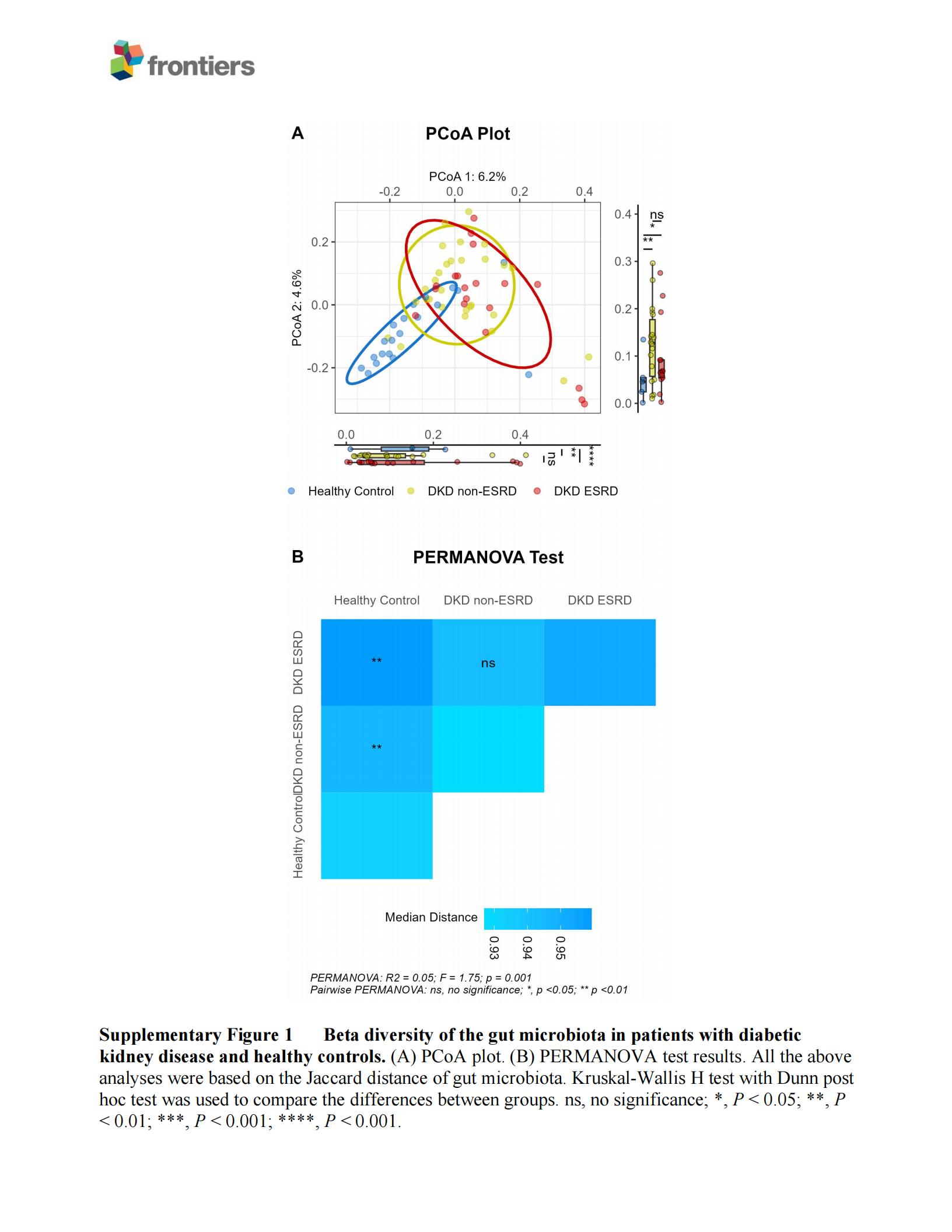

Supplement: Supplementary file 1 [file Image_1.jpeg]

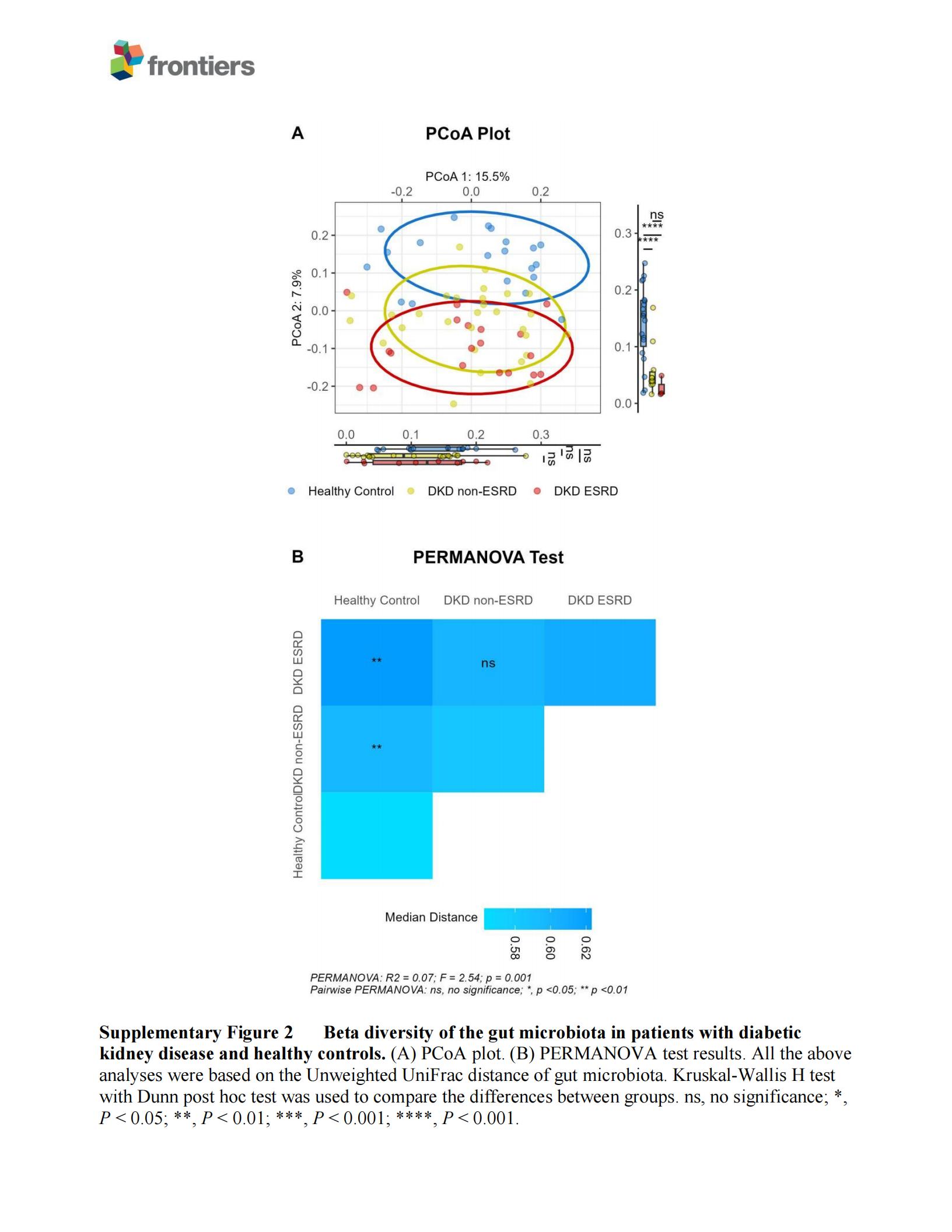

Supplement: Supplementary file 2 [file Image_2.jpeg]

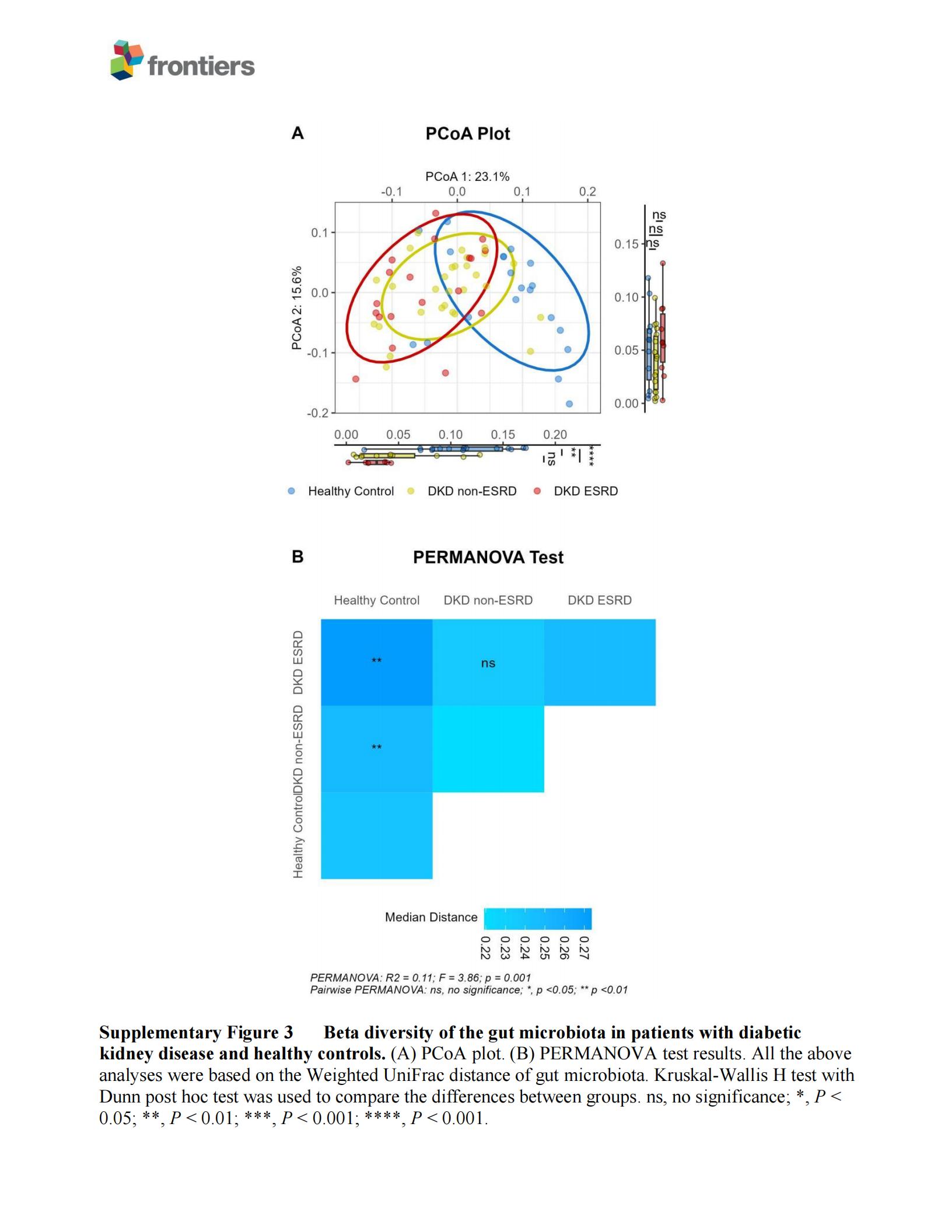

Supplement: Supplementary file 3 [file Image_3.jpeg]
